# The association between gray matter volume in the hippocampal subfield and antidepressant efficacy mediated by abnormal dynamic functional connectivity

**DOI:** 10.1038/s41598-024-56866-w

**Published:** 2024-04-18

**Authors:** Changxiao Kuai, Jiayong Pu, Donglin Wang, Zhonglin Tan, Yan Wang, Shao-Wei Xue

**Affiliations:** 1 Center for Cognition and Brain Disorders, The Affiliated Hospital of Hangzhou Normal University, No. 2318, Yuhangtang Rd, Hangzhou, 311121 Zhejiang Province People’s Republic of China; 2 Institute of Psychological Science, Hangzhou Normal University, Hangzhou, Zhejiang Province People’s Republic of China; 3 Zhejiang Key Laboratory for Research in Assessment of Cognitive Impairments, Hangzhou, Zhejiang Province People’s Republic of China; 4 Affiliated Mental Health Center & Hangzhou Seventh People’s Hospital, Zhejiang University School of Medicine, Hangzhou, Zhejiang Province People’s Republic of China

**Keywords:** Cognitive neuroscience, Computational neuroscience, Diseases of the nervous system

## Abstract

An abnormality of structures and functions in the hippocampus may have a key role in the pathophysiology of major depressive disorder (MDD). However, it is unclear whether structure factors of the hippocampus effectively impact antidepressant responses by hippocampal functional activity in MDD patients. We collected longitudinal data from 36 MDD patients before and after a 3-month course of antidepressant pharmacotherapy. Additionally, we obtained baseline data from 43 healthy controls matched for sex and age. Using resting-state functional magnetic resonance imaging (rs-fMRI), we estimated the dynamic functional connectivity (dFC) of the hippocampal subregions using a sliding-window method. The gray matter volume was calculated using voxel-based morphometry (VBM). The results indicated that patients with MDD exhibited significantly lower dFC of the left rostral hippocampus (rHipp.L) with the right precentral gyrus, left superior temporal gyrus and left postcentral gyrus compared to healthy controls at baseline. In MDD patients, the dFC of the rHipp.L with right precentral gyrus at baseline was correlated with both the rHipp.L volume and HAMD remission rate, and also mediated the effects of the rHipp.L volume on antidepressant performance. Our findings suggested that the interaction between hippocampal structure and functional activity might affect antidepressant performance, which provided a novel insight into the hippocampus-related neurobiological mechanism of MDD.

## Introduction

Major depressive disorder (MDD) is a common but very debilitating condition which is usually characterized by depressed mood or loss of interest along with other symptoms, including significant changes in weight or appetite, hypersomnia or insomnia, retardation or psychomotor agitation nearly every day, and feelings of worthlessness or excessive or inappropriate guilt^1^. As the prevalence is growing over time, MDD is ranked as the main cause of the burden of disease worldwide by WHO^2^. The nature of the clinical disturbance may give clues during studying the neuropathology of MDD, typified by a wide-ranging but unconclusive distribution of brain alterations involved in cognitive dysfunction, emotion processing and physical symptoms^3^.

Although antidepressant medication (ADM) has been recommended as the first-line treatment for MDD patients, the treatment remission rate is only 30–40%^4^. In most circumstances, clinicians need several weeks to examine the treatment response and determine whether to change the medical treatment plan in clinical practice, which would prolong the suffering of MDD patients^5,6^. The treatment of MDD can be characterized by 3 phases: acute phase (0–3 months), continuation phase (4–9 months), and maintenance phase (≥ 1 year)^7^. Clinically, psychiatrists strive to control MDD patients' depressive symptoms within 3 months to achieve clinical cure as much as possible and promote the recovery of function to the pre-disease level. The outcome and prognosis of the disease depend on the curative in the acute phase, and 3-month is the critical time point for treatment. Therefore, exploring and predicting the improvement after 3 months of antidepressant treatment will help psychiatrists select suitable treatment schemes for the continuation phase and the maintenance phase. It will also improve the treatment remission rate of patients with MDD. A previous meta-analysis based on studies over the past 15 years has revealed that some different biomarkers have already shown their capacity to predict response to antidepressant treatment. Compared to cognition, proteins, electrophysiology or genetics, neuroimaging measures exhibited a more desirable predictive manner, and it might have reminded clinicians to refer flexibly and employ alternative treatment procedures or combination therapies on those patients who were insensitive to first-line antidepressants^8^.

The hippocampus is a pivotal brain region that participates in a series of cognitive and affective functions^9,10^. A previous meta-analysis of MDD studies found that hippocampal dysfunction was related to emotional symptoms and memory impairment^11^. This unique role of the hippocampus on emotional memory, especially negative emotional memory, is not available in other brain regions. Meanwhile, a review using ultrahigh-field imaging indicated that the hippocampus exhibited more volumetric and structural connectivity changes than the amygdala in MDD^12^. Therefore, we started from the hippocampus as a key focus rather than from other areas. Previous studies^13,14^ have shown that long-term exposure to high levels of glucocorticoids could increase neuronal cell death in the hippocampus and cause the hippocampus to shrink and lead to impairments in hippocampal synaptic plasticity in MDD patients, eventually resulting cognitive impairments related to the pathology^15^. Previous magnetic resonance imaging (MRI) research has revealed that a smaller hippocampal volume and anomalous hippocampal functional connectivity (FC) might cause damage in emotion regulation and memory^16–18^, especially those memories related to negative emotions in MDD patients^19,20^. These findings indicated that the abnormal structure and function of the hippocampus might be key components of the physiopathology of MDD. Using the hippocampus neuroimaging index as a biomarker, a previous study demonstrated that a ‘less abnormal’ hippocampal volume could predict a quicker response to antidepressants and a better treatment remission^21^. In animal research, even after short-term treatment, antidepressant treatment has been revealed to reverse impaired neurogenesis and neuroplasticity in the hippocampus^22,23^. However, because antidepressant treatment can significantly increase hippocampal volume and the effects might persist even after a washout period^21^, as well as the complex relationship between hippocampal volume alteration and illness duration, it is complicated and difficult to use only hippocampal volume to predict the antidepressant efficacy. Increasingly studies have shown that the aberrant hippocampal FC has the ability to predict a poor antidepressant response after the acute phase treatment. One recent research reported the predictive value of hippocampal FC for the antidepressant treatment response after 2-week treatment^24^, and another study indicated that the hippocampal functional connectivity patterns of brain regions between and within networks might play a pivotal role in identifying a favorable response for the 8 weeks’ treatment for patients with MDD^25^. A recent study found significant negative correlations between hippocampal volume and hippocampal FC with the inferior parietal lobe (IPL) and thalamus^26^ and it is more reliable to use FC and volume together to predict medical efficacy.

However, previous studies mainly used static functional connectivity^27,28^. Using this approach, an implicit assumption is that FC remains throughout the entire duration of the MRI scan. These time-averaged FC metrics would ignore the dynamic characteristic of MRI signals and the underlying temporal variations of FC which may supply additional information about brain activity^29^. This temporal fluctuation of FC is referred to as dynamic functional connectivity (dFC). By using techniques of dynamic analysis, we can track the real-time activity changes in brain connectivity across different brain states. Recent evidence has demonstrated that dynamic functional connectivity can provide new information on temporal variability of rsFC and also recurring patterns of rsFC over time^30,31^. Currently, in the fields of neuroscience and mental illness, more and more studies applied dFC to depict the brain alterations in some neuropsychiatric diseases including attention deficit hyperactivity disorder, schizophrenia, especially major depressive disorder^32,33^.

The structural complexity and functional diversity of the hippocampus demonstrate the existence of different structural and functional subdivisions within this structure^10,34^. Many studies have showed that separate hippocampal subregions have different effects on different emotional and cognitive activities^35–37^. A principal theory of functional differentiation within the hippocampus suggests that the functional role of the hippocampus varies along its longitudinal axis, that is, the anterior hippocampus is responsible for emotional reactions and the posterior hippocampus contribute to cognitive functions^38^. There is substantial evidence demonstrating that the anterior portion of the hippocampus, namely the rostral hippocampus, is highly involved in recollection of previous experience events and retrieval of unpleasant experiences, as well as modulating affective processing in particular of sadness and fear. Whereas the posterior part of the hippocampus, namely the caudal hippocampus, is related to perceptual functioning. Furthermore, encoding in episodic memory uses more anterior regions of the hippocampal formation, whereas spatial navigation utilizes more posterior regions, which suggests a specific role of the anterior hippocampus in remembering emotion-related stimuli. Evolutionary and functional neuroimaging evidence suggests that the right and left hippocampus have functional differences. Most theories suggest that the right hippocampus is more related to nonverbal and spatial functions, while the left hippocampus is more associated with verbal memory functions^39,40^. One recent research illustrated that using specific subfields of the hippocampus as neuroimaging biomarkers may improve the ability to choose the best first-time treatment strategy for newly diagnosed patients with MDD^21^. Therefore, we conjectured that, compared to using the unsubdivided whole hippocampus, employing the left rostral hippocampus might have better predictive value for antidepressant efficacy in patients with MDD.

MDD is associated with smaller volume or abnormal connectivity of the hippocampus. However, the association between hippocampal structural and functional abnormalities and the impact on clinical treatment remain unclear, which may be a important research direction for the pathogenesis of MDD. Studying how the volume and dynamic functional connectivity of the left rostral hippocampus relate to the outcomes of 3-month antidepressant treatment in patients with MDD will help to reveal the role of the structure and function of the hippocampal subregion in the pathogenesis and treatment of MDD, and may provide us with more effective treatment strategies and intervention methods. In the broader context of this field, it can help us to gain a deeper understanding of the pathophysiology of MDD and provide theoretical support for the development of more effective treatments.

In the present research, we were to characterize the relationships among the volume of the left rostral hippocampus, the dynamic functional connectivity of the left rostral hippocampus and 3-month antidepressant pharmacotherapy outcomes for MDD patients. We hypothesized that the dFC of the left rostral hippocampus would be associated with both the volume of the left rostral hippocampus and the antidepressant efficacy, and would mediate the relationship between them. The primary goal was to search for novel evidence for the application of neuroimaging techniques in the prediction of treatment efficacy and to enrich more individualized therapy proposals for MDD patients.

## Methods

### Participants

The demographic and clinical characteristics of the participants were summarized in Table 1. Thirty-six MDD patients (age: 28.03 ± 10.20 years; gender: 26 females/10 males) and forty-three healthy controls (age: 29.42 ± 12.56 years; gender: 27 females/16 males) were included in the present study. The interviews and diagnoses of MDD patients were completed by certified psychiatrists from the Affiliated Mental Health Center of Zhejiang University School of Medicine by the Mini Neuropsychiatric International Interview (MINI) following the criteria of Diagnostic and Statistical Manual of Mental Disorders, Fourth Edition (DSM-IV). The severity of depressive symptoms was evaluated by using the 24-item Hamilton Depression Rating Scale (HAMD) in subjects including MDD patients and HC. The participants who met the following criteria were excluded from this study: (1) having severe suicidal tendencies; (2) having neurological or medical illness; (3) having substance dependence; (4) pregnancy or breastfeeding; (5) having some contraindications such as with metallic implants. Following a complete written and oral explanation of this study, each subject provided written consent to participate in the study. All research procedures were performed in accordance with the Declaration of Helsinki on Ethical Principles and were approved by the local Institutional Review Boards of Hangzhou Normal University.

**Table 1:** Demographic and clinical data.

Characteristics	MDD (mean ± SD)	HC (mean ± SD)	*p *value
Sex (male/female)	36 (10/26)	43 (16/27)	0.374^a^
Age (years)	28.03 ± 10.20	29.42 ± 12.56	0.595^b^
Baseline HAMD	27.78 ± 6.70	1.35 ± 1.38	< 0.001^b^
3-month HAMD	11.42 ± 7.09		
Duration of illness (months)	9.36 ± 14.96		
First	25		
Recurrence	11		
On-medication SSRIs (n patients)	36		

The data were presented as the mean ± standard deviation.

*HC* healthy control, *MDD* major depressive disorder; *HAMD* Hamilton Depression Rating Scale, *SSRIs* selective serotonin reuptake inhibitors.

^a^The *p* value was obtained by a chi-square test;

^b^The *p *value was obtained by a two-tailed two-sample t-test.

### Antidepressant treatment

The longitudinal data were collected from patients with MDD before and after 3 months of antidepressant pharmacotherapy with selective serotonin reuptake inhibitors (SSRIs). The medication dosages were prescribed and adjusted by the treating psychiatrists according to routine clinical practice and followed the recommended dosages ranges. After a 3-month antidepressant treatment, all MDD patients underwent an MRI scan and psychiatric assessments including HAMD evaluation again. HC participants did not take any medicine and only received the baseline MRI scan. The remission rate (RR%) was defined as (Baseline HAMD scores – 3-month HAMD scores)/Baseline HAMD scores. It should be noted that this definition of remission, with RR%, differs from the well-known definition based on HAMD scores of less than 10 or 7.

### MRI data acquisition

The imaging data of all participants were obtained using a 3.0 T Discovery MR 750 scanner (General Electric, Waukesha, WI, USA) at the Affiliated Hospital of Hangzhou Normal University. Participants were instructed to keep their eyes closed and remain still without focusing on any specific thoughts while staying awake during the scanning process. To minimize motion artifacts, a pair of stabilizers was utilized to immobilize the participants' heads. Functional images were acquired in an interleaved manner using a T2*-weighted gradient-echo EPI pulse sequence. The acquisition parameters were as follows: repetition time (TR) = 2000 ms, echo time (TE) = 22 ms, flip angle = 77°, field of view (FOV) = 240 × 240 mm^2^, matrix = 96 × 96, isotropic spatial resolution of 2.5 mm with 42 slices and a total of 240 volumes. High-resolution T1-weighted anatomical images were obtained in sagittal orientation for visualization and localization purposes using a fast spoiled gradient echo sequence (3D FSPGR): TR = 9 ms, TE = 3.66 ms, flip angle = 13°, field of view (FOV) = 240 × 240 mm^2^, matrix = 300 × 300, 0.8 mm isotropic voxels, 176 slices without interslice gap.

### MRI preprocessing

The T1-weighted brain structural images were pre-processed and examined using CAT12 (Computational Anatomy Toolbox 12, http://www.neuro.uni-jena.de/cat/) based on the SPM12 software (http://www.fil.ion.ucl.ac.uk/spm/software/spm12). CAT12 is a widely used program for performing voxel-based morphometric (VBM) analysis. In the VBM analysis, we estimated a non-linear deformation field that can best overlays the tissue probability maps on individual participant’s MRI images. The original T1-weighted images were segmented into three tissue components, including the gray matter (GM), the white matter (WM), and the cerebral spinal fluid (CSF). The overall tissue volume and total intracranial volume were calculated in the native space. Afterwards, the segmented GM images and WM images of all participants were used to create a study-specific template by using DARTEL (Diffeomorphic Anatomical Registration using Exponentiated Lie algebra toolbox). The Montreal Neurological Institute (MNI) standard brain template was adopted to achieve normalization of the standard space using modulation, the voxel size for normalized images was 1.5 × 1.5 × 1.5 mm^3^. Finally, the modulated GM tissue segments were smoothed with an 8-mm full width at half maximum (FWHM) isotropic Gaussian kernel. We obtained the smoothed and modulated GM images for each participant to be used in further statistical analysis. In order to exclude the influence of total intracranial volume on statistics, we calculated not only the absolute volume in cubic centimeters, but also the relative volume obtained by dividing the absolute volume by the total cerebral volume (TCV) including the cerebrospinal fluid volume and use them in subsequent analysis.

The processing of original rs-fMRI image data was conducted using a combination of the DPABI software (for Data Processing & Analysis of Brain Imaging, http://rfmri.org/dpabi) and custom code written in MATLAB (The MathWorks, Inc., Natick, MA, United States). The first ten functional volumes of participants were discarded to allow for magnetization equilibration effects and the adaptation of the participants to the circumstances. The remaining images were corrected for time delay between slices and then realigned to the first volume for head motion correction. Throughout the entire scanning process, all participants had a maximum displacement of less than 2 mm in the x-, y-, or z-axes and an angular motion of less than 2°. To further control the confounding effects of head motion, the framewise displacement (FD) across time points was calculated for further analysis. The 3D T1-based transformation was used to perform the spatial normalization of the functional images. The 3D T1 images were co-registered to mean functional images. The corrected images were spatially normalized into the standard stereotactic space of the Montreal Neurological Institute using the transformation derived from T1 segmentation and resampled into 3-mm isotropic voxels. To further reduce the influences of confounding factors, we also regressed out the following sources: six motion parameters, white matter signal and cerebrospinal fluid (CSF) signal. After band-pass filtering (0.01–0.1 Hz), we used the cubic spline method, specifically removing “bad” time points from the time series with an FD threshold of 0.5 mm, to reduce the effects of head motion^41^. All participants’ images were visually inspected for accuracy of segmentation, registration, skull stripping, and cortical surface reconstruction.

### Sliding window dFC analysis of the left rostral hippocampus

**Figure 1:**
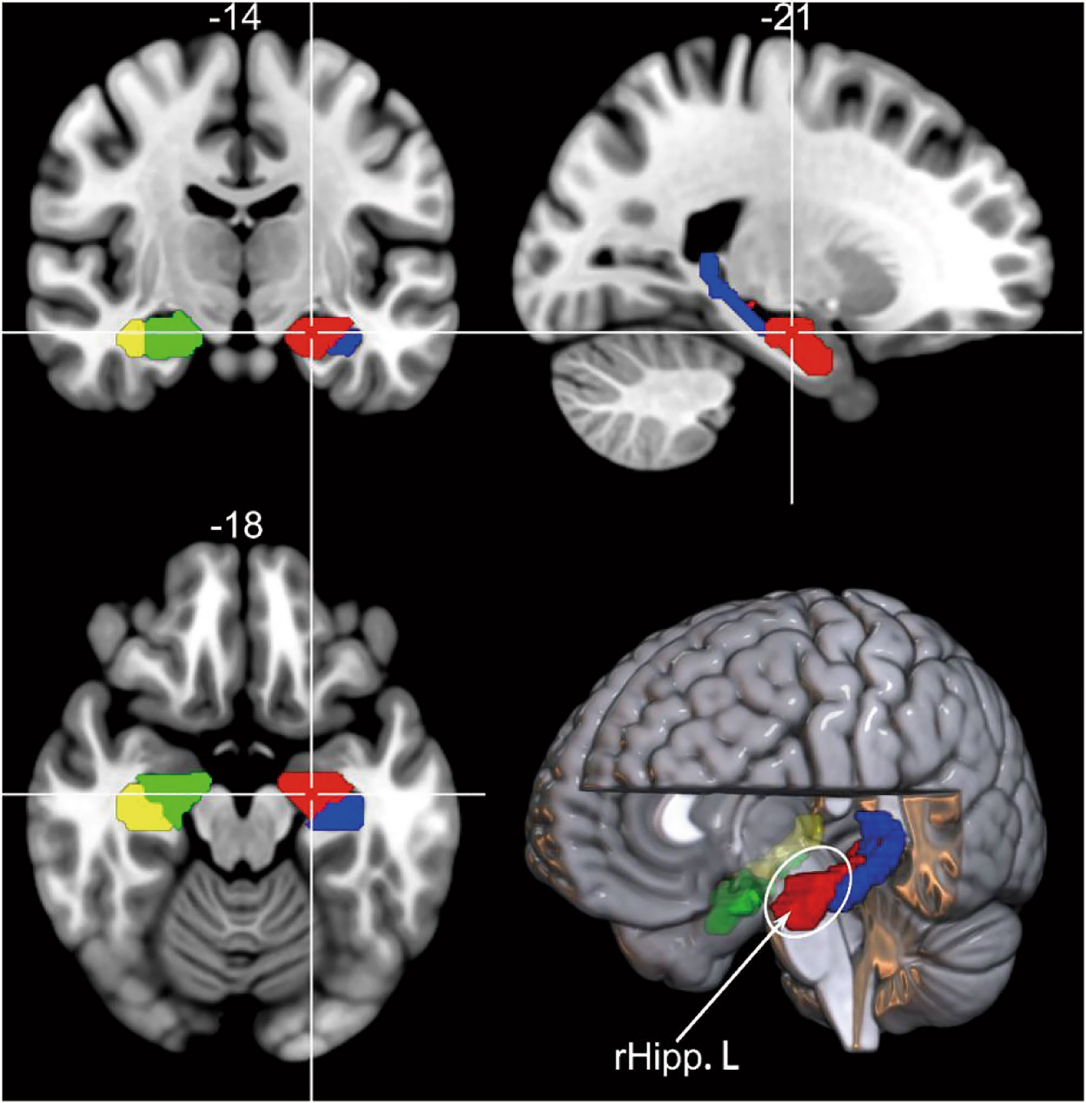
Four hippocampal subregions. The red cluster marked is our region of interest. Abbreviations: rHipp.L, the left rostral hippocampus.

Following a brain template from the Human Brainnetome Atlas Project (http://atlas.brainnetome.org), the hippocampus in each hemisphere was divided into 4 subregions: the left rostral hippocampus, the right rostral hippocampus, the left caudal hippocampus, and the right caudal hippocampus (Fig. 1). To obtain the whole-brain dFC map of the left rostral hippocampus, we adopted a sliding-window approach for each subject. We used a window created by convolving a rectangle with a Gaussian (σ = 3 TRs) and iteratively sliding each window by 1 TR (2 s). In a Gaussian window, the data at the beginning and the end of the window were downweighed. By setting the window size to 22 TRs (44 s), we obtained 209 consecutive windows for each participant. To reduce low-frequency excursions and respiratory or cardiac noise at high frequency, the rs-fMRI data of each window were temporally bandpass filtered ([0.01–0.1 Hz]). Within each sliding window, for each seed region of interest (ROI), we calculated the Pearson correlation coefficient between the mean rs-fMRI signal of the left rostral hippocampus and signals of other voxels. The resulting correlation coefficients were converted to z-scores using the Fisher r-to-z transformation to improve normality. Therefore, we obtained 209 z-score maps for each participant, representing the whole-brain dFC fluctuation for the left rostral hippocampus. Specifically, the dFC was estimated by calculating the mean of the z maps across the 209 windows, and the resulting dFC maps were then z-standardized.

### Statistical analysis

The two-sample *t*-test was employed to analyze differences in age and HAMD scores, and the chi-square test was used to assess gender differences between the MDD and HC groups. A paired sample *t*-test was conducted to compare the baseline and post-intervention dFC or volume within the MDD group. Additionally, we performed a two-sample *t*-test to compare the difference in dFC or volume between MDD patients and HCs, with gender, age, and head movement of all participants regressed as covariates. To control for multiple comparisons, FWER corrections (family-wise error rate) with Gaussian random field theory were employed with the voxel threshold set to *p* < 0.001 and the cluster threshold set to *p* < 0.0125 (0.05/4). Furthermore, we carried out Pearson correlation analysis in the MDD group to explore the relationship between the neuroimaging findings (dFC and volume) and clinical characteristics (HAMD).

To investigate whether the influence of the left rostral hippocampal volume on antidepressant efficacy was mediated by its abnormal dFC, a mediation analysis based on a standard three-variable mediation model was performed. The independent variable was the relative volume of the left rostral hippocampus, the mediator was its dFC, and the dependent variable was the antidepressant efficacy after a 3-month treatment. The significance of the mediation effect was assessed using bootstrapping method and bias-corrected 95% confidence intervals (CI) in PROCESS macro (V4) for SPSS (version 25; IBM SPSS Inc.). A confidence interval that does not contain zero means that there exists a significant mediation effect for the proposed mediating factor.

### Ethical approval

The studies involving human participants were reviewed and approved by the Institutional Review Boards of Hangzhou Normal University.

### Informed consent

The patients/participants provided their written informed consent to participate in this study.

## Results

### Altered dynamic functional connectivity pattern of the left rostral hippocampus in MDD

**Figure 2:**
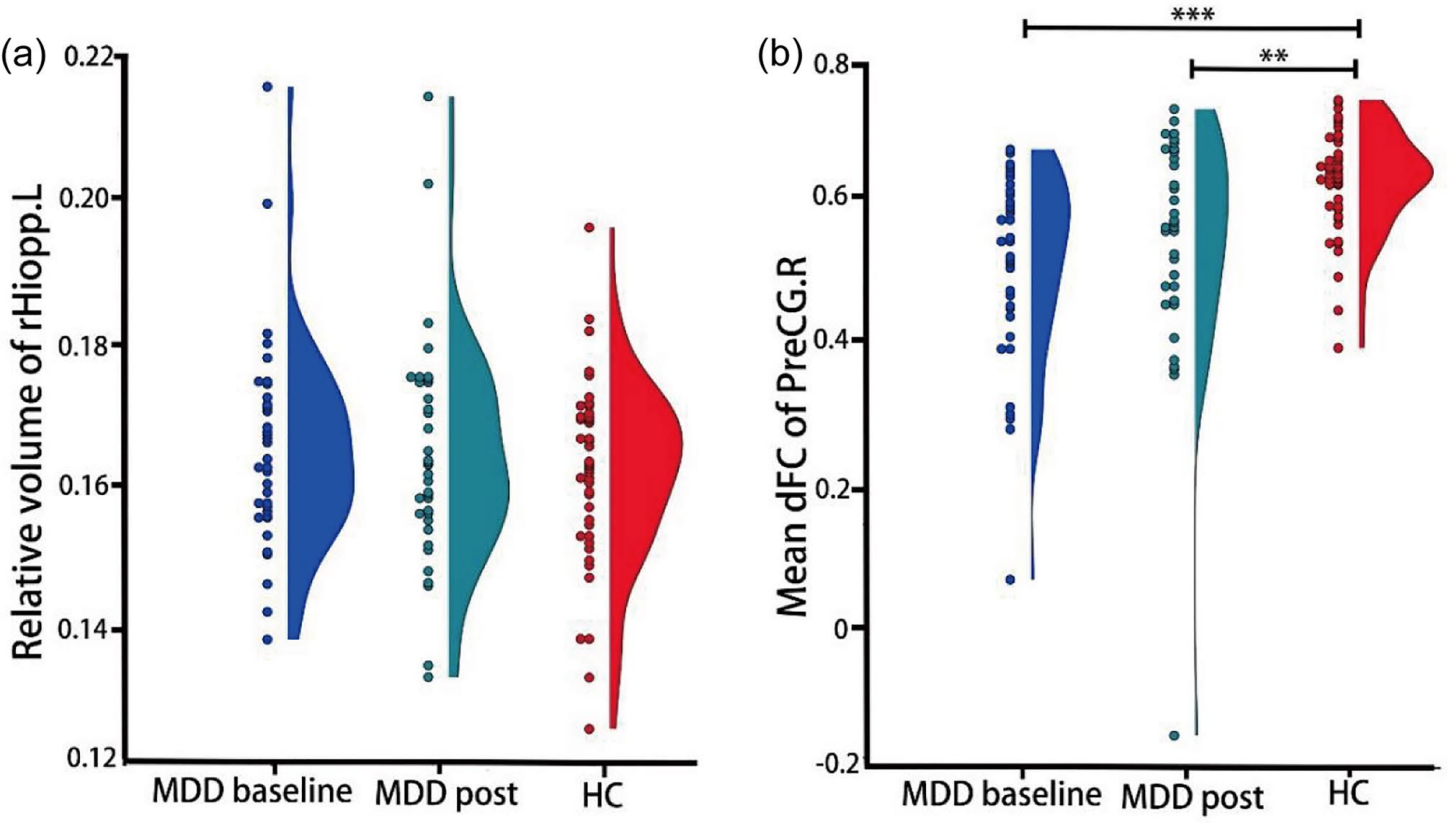
(**a**) Between-group differences of the relative volume of rHiopp.L; (**b**) Between-group differences mean dFC of rHipp.L with PreCG.R. Abbreviations: MDD, major depressive disorder; rHipp.L, the left rostral hippocampus; dFC, dynamic functional connectivity; PreCG.R, the right precentral gyrus.

Compared with HC, patients with MDD showed statistically significant decreased dFC of the left rostral hippocampus (rHipp.L) with three brain regions including the right precentral gyrus (PreCG.R, *p* < 0.0125, FWE-corrected; peak MNI coordinates: x = 48, y = − 9, z = 57, t = − 5.03, cluster size = 374 voxels), postcentral gyrus (PoCG.L, *p* < 0.05) and left superior temporal gyrus (STG.L, *p* < 0.05). Thereinto, the case–control comparison results of the dFC between the rHipp.L and PreCG.R were shown in Fig. 2. There were significant differences in the dFC of the rHipp.L with the PreCG.R between MDD patients at baseline and HC (*p* < 0.001), as well as between MDD patients after 3-month antidepressant treatment and HC (*p* < 0.01). There were no significant differences in the volume of the rHipp.L between MDD and HC (*p* > 0.05).

### Mediation analysis of hippocampal volume and function for antidepressant efficacy

**Figure 3:**
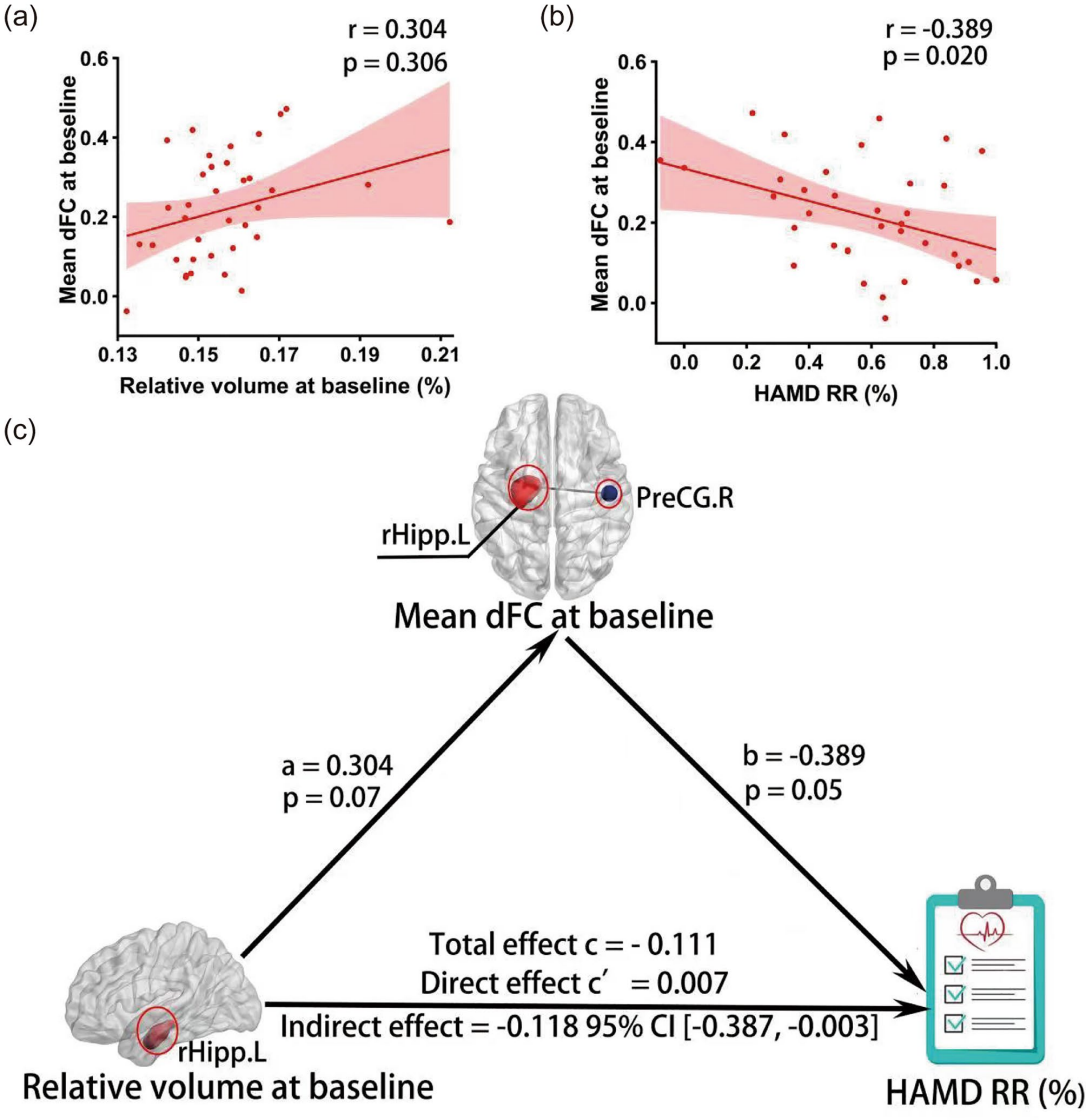
Correlation and mediation analysis. (**a**) The mean dFC of the rHipp.L with PreCG.R at baseline was correlated with the relative volume of rHiopp.L; (**b**) The mean dFC of the rHipp.L with PreCG.R at baseline was correlated with HAMD remission rate; (**c**) The mean dFC at baseline between the rHipp.L and PreCG.R significantly mediates the relationship between the relative rHipp.L volume at baseline and HAMD RR [Indirect effect c’ = − 0.118 95% CI [− 0.387, − 0.003], Direct effect c’ = 0.007, Total effect c = − 0.111]. Abbreviations: dFC, dynamic functional connectivity; rHipp.L, the left rostral hippocampus; PreCG.R, the right precentral gyrus; HAMD, Hamilton Depression Rating Scale; RR, remission rate; CI, confidence interval.

As shown in Fig. 3, the dFC between the rHipp.L and PreCG.R demonstrated statistically significant associations with the relative volume of the rHipp.L (r = 0.304, *p* = 0.07) and HAMD remission rate (r = − 0.389, *p* = 0.05) in MDD respectively. Further mediation analysis found that this dFC of the rHipp.L could significantly mediate the relationship between the relative volume of the rHipp.L and HAMD remission rate in MDD patients. Our results revealed that when the rHipp.L relative volume was examined as a possible predicting factor, the total effect c = − 0.111, the direct effect c’ = 0.007 (95% CI [− 580.12, 604.2]), the indirect effect c–c’ = − 0.118 (95% CI [− 0.387, − 0.003]) the dFC between rHipp.L and PreCG. L was a significant mediator in the relationship between the relative volume of rHipp.L and antidepressant efficacy after 3-month medical treatment in MDD patients (Fig. 3).

## Discussion

The present study is aimed to characterize the relationship among antidepressant responses, hippocampal structure and function. We mainly focused on the effect of the left rostral hippocampal region on antidepressant pharmacotherapy responses for MDD. The results showed that the dFC of the left rostral hippocampal subregion mediated the relationship between the hippocampal volume and antidepressant efficacy. The findings advanced our understanding of how the hippocampus affects the treatment effects and provided potential biomarkers for treatment prediction and evaluations in MDD.

Many studies have provided consistent evidence of the importance of the hippocampus in the development or treatment of MDD^18,21,42^. Hippocampal volume is proposed as a potentially useful neuroimaging biomarker of treatment sensitivity to antidepressant. One recent research^43^ revealed that increased hippocampal tail volume could predict depression status and remission to antidepressant medications in MDD, and another research^44^ reported smaller left and right hippocampal volumes in patients with MDD. These findings suggested the importance of using VBM analysis to explore the volume of specific hippocampal subregions. The dFC provided a new tool for exploring the neurophysiological mechanism of MDD. One previous study^45^ has also demonstrated altered dFC variability in the medial prefrontal cortex (mPFC) and large-scale functional networks in MDD patients. These findings prompted us that the combined use of VBM and dFC analysis might provide novel understandings of MDD pathology. Therefore, VBM analysis and seed-based dFC analysis were two interrelated analytical methods employed in this study.

In our present research, MDD patients showed lower dFC in the PoCG.L, STG.L and PreCG.R when using the rHipp.L as a seed compared with HC. The next correlation analysis showed that the dFC between the rHipp.L and PreCG.R was significantly negatively correlated to the remission rate of HAMD, which confirmed previous research. The precentral gyrus is the site of the main somatosensory cortex of the human brain that is highly involved in some pivotal cognitive activities, including working memory^46^, implicit learning^47^ and motor learning^48^. The abnormalities in the structure and function of the precentral gyrus in MDD patients have been revealed in many neuroimaging studies^49–51^. In addition, superior control-related activation of the precentral gyrus is associated with suicide risk in MDD patients^52,53^. Therefore, we have reason to assume that the aberrant dFC between rHipp.L and PreCG.R leads to abnormally excessive suicidal beliefs and suicidal behaviors in MDD patients. Furthermore, the gray matter volume of the precentral gyrus in MDD patients may be related to avoidance motivation, which is one of the most significant characteristics of MDD patients^54^. Most importantly, this was found to be associated with negative attribution bias^55^. Internal attributions of events, where the self is viewed as an active intentional agent, involve PreCG. Aberrant activation of PreCG may lead MDD patients to use fewer “self-service” attribution manners than HC, which may explain the feelings of worthlessness or excessive or inappropriate guilt in MDD patients. According to previous studies^56,57^, treatment with escitalopram for 8 weeks was found to relate to the FC abnormalities in the hippocampus and precuneus, and SSRI administration was also reported to decreased the FC of the hippocampus with the prefrontal cortex. Our findings, together with previous studies, suggested that the left hippocampus might be a target for rapid antidepressant action, which requires further study for verification.

Further correlation analysis revealed that the dFC between PreCG.R and rHipp.L was significantly positively correlated to the relative volume of rHipp.L. Two hypotheses have been proposed to explain the hippocampal volume: the neuroplasticity hypothesis and the neurogenesis hypothesis^13^. The neuroplasticity hypothesis addresses that the hippocampal volume decreased due to the shortening of dendrites and a reduction in the number and density of spines. The Neurogenesis hypothesis resolves the issue that the hippocampal volume is decreased by the decrease of neurogenesis in the hippocampal dentate gyrus. These two hypotheses assume that, whether from the perspective of morphological changes of hippocampal neurons or from the perspective of the hippocampal dentate gyrus, as the hippocampus shrinks in patients with MDD, the connectivity of the hippocampus also decreases, which is verified by our results.

It was worth noting that there were no significant differences between the relative volume of rHipp.L in MDD patients at baseline, after 3-month antidepressant treatment, and HC. Although this appears to be a consensus regarding the abnormalities of the hypothalamic–pituitary–adrenal (HPA) axis observed in MDD patients, and the hippocampus has been suggested as a suprahypothalamic regulator involved in the negative feedback control of cortisol^58^, higher levels of cortisol may induce neuronal damage in the hippocampus, leading to hippocampal shrinkage. But changes of the hippocampal volume in MDD patients are still a topic of debate. Although smaller hippocampal volume in MDD relative to HC was more frequently reported, some studies have come up with different results. One recent research^43^ revealed that larger hippocampal tail volume was associated with both a diagnosis of MDD and remission to antidepressant drugs, another study^59^ found no differences between the hippocampal volume of nonpsychiatric healthy controls and MDD patients. In our present research, no significant differences were found. This may be the result of a mixed effect of many factors, such as the long illness duration, medication effect and MDD patients’ age. This complicated relationship should be further examined in the future. Fewer neurons or glia may result in smaller hippocampal volume in MDD patients. However, the density of CA1 pyramidal neurons increases with the duration of illness, and the number of dentate gyrus granule cells and glial cells increases with age, leaving no significant changes in hippocampal volume. Therefore, we considered that the changes in hippocampal volume was the result of a mixed effect of many factors such as the long illness duration, medication effect and MDD patients’ age, and this complicated relationship should be further examined in the future.

After the mediation analysis, the dFC was found to significantly mediate the relationship between the relative volume of rHipp.L and antidepressant efficacy. Because we found abnormal dFC at baseline in MDD patients, and no significant differences in rHipp.L volume were found in group comparisons. It is reasonable to assume that the dFC abnormalities might precede structural abnormalities, suggesting that these imaging phenotypes may indicate distinct stages of cognitive impairment in MDD patients. One study indicated that antidepressant drugs may reverse both the neurogenesis modifications and the impaired neuroplasticity in the hippocampus, such as loss of glial cells and dendritic atrophy^23^, which may normalize the shrunken hippocampus. More importantly, many studies^24,60,61^ have proved that after different phases of antidepressant treatment, even only two weeks, the FC using the hippocampus as a seed improved to varying degrees. We proposed that such a normalization process in the hippocampal connectivity may lead to a beneficial impact on emotional regulation functions and cognitive ability in MDD patients. At the same time, these findings further proved the importance of using dFC as a mediation variable to explore the relationship between the specific hippocampal subregion volume and antidepressant efficacy.

Several limitations of our present research should be solved. Firstly, the antidepressant treatment effect in the present study was for pharmacological treatment and the treatment effect of other treatment protocols is worthy of being studied carefully. Secondly, the sample size of MDD patients was relatively small and there was a gender ratio discrepancy between MDD and HC participants, which might reduce the generalizability of the work, and our study should be regarded as a pilot study. Future longitudinal studies with large sample sizes and patients with various clinical features are needed to recapitulate the results of our study before they can be applied in clinical practice.

## Conclusions

The present study revealed the abnormal dFC between the PreCG.R and rHipp.L in MDD patients, although no volume changes have yet been detected. Furthermore, the dFC mediated the relationship between the rHipp.L volume and antidepressant efficacy, suggesting that there exists temporal dynamics of hippocampal functional and structural alterations during the disease progression of MDD patients. Most importantly, our research showed that a combination of volume and abnormal dFC yielded higher discriminative value in predicting antidepressant efficacy in MDD patients compared with either alone. Our present research revealed the importance of the volume and dFC of specific hippocampal subfield as a neuroimaging biomarker, and provided new evidence supporting the application of neuroimaging techniques in the treatment outcome prediction, thus guiding a more individualized treatment strategy for MDD patients.

## Data Availability

The data that support the findings of this study are available from the corresponding author upon reasonable request.
